# Optimizing energy balance in youth: reducing sugar-sweetened beverages and screen time

**DOI:** 10.1093/pch/pxae107

**Published:** 2025-05-07

**Authors:** Hannah M Murphy, Kelsey M Stanford, Scott V Harding

**Affiliations:** Department of Biochemistry, Faculty of Science, Memorial University, St. John’s, NL, Canada; Department of Biochemistry, Faculty of Science, Memorial University, St. John’s, NL, Canada; Department of Biochemistry, Faculty of Science, Memorial University, St. John’s, NL, Canada

**Keywords:** *Energy*, *Balance*, *Youth*, *Sugar-sweetened beverages*

## Abstract

The high prevalence of children and adolescents living with obesity, as well as the multitude of associated risks, stress the need for refined weight management strategies for this population. While an overarching lifestyle intervention may be an ideal way to improve energy balance, more practical recommendations would likely improve adherence rates. This review set out to investigate sugar-sweetened beverage consumption and screen time as potential lifestyle targets to reduce paediatric obesity. We report strong evidence that both sugar-sweetened beverage consumption and screen time influence childhood obesity directly, as well as through interactions with sleep patterns. Potential mechanisms are discussed with focus on the energy balance framework of obesity. We also present methodological considerations to improve applicability and consistency of future studies.

The percentage of overweight youth has increased considerably in the past several decades and in 2016 the World Health Organization (WHO) estimated that over 340 million children and adolescents were living with overweight or obesity (1). The negative effects of obesity on health are widespread, with complications and comorbidities within the body’s endocrine, cardiovascular, gastrointestinal, musculoskeletal, nervous, integumentary, pulmonary systems, and psychological well-being (2–4). Children and adolescents with obesity are of particular concern because, as weight management issues often persist into adulthood, being obese early in life greatly increases the likelihood of obesity later in life (5). Furthermore, childhood and adolescents with obesity has been linked to increased risk for future premature mortality (4,6). These risks, along with the alarmingly high prevalence of obesity in this age group, stress the need for promotion of weight management strategies in public health.

The energy balance framework of obesity considers imbalances that occur due to differences in energy intakes and expenditures, leading to subsequent changes in body weight (3,7). Energy intake encompasses the macronutrients we consume in our diet, while energy is expended through resting metabolic rate, thermic effect of food, illness and injury repair, and our physical activity levels (7). Circadian rhythms, which are 24-h oscillations in metabolism, physiology, and behaviour, can have a great impact on this balance (8). The hypothalamic suprachiasmatic nucleus is considered the master regulator of the rhythms; however, evidence suggests that cells in all body systems contribute to the patterns (9). Circadian rhythms have been shown to interact with most metabolic processes: The secretion of leptin, cortisol, and insulin, as well as glucose tolerance, are all elements of metabolism that have been shown to be influenced by circadian cycles (10). As sleep cycles are a prominent circadian rhythm, insufficient sleep can disrupt balances and lead to disturbed metabolic function (11). This idea that sleep patterns may mediate energy balance, makes adequate sleep an important consideration in weight management.

Challenges arise when devising public health strategies to combat overweight and obesity as multiple factors contribute to the problem, making the epidemiology difficult to delineate (12). In line with this interplay of factors that influence obesity, overall lifestyle change is the gold standard recommendation for both the prevention and intervention in overweight and obesity (3). However, previous reports have identified time constraints, overwhelming effort, and financial struggles as barriers to successful lifestyle modification programs (13). Therefore, to facilitate participation and ensure adherence to such programs, we must identify specific targets whereby small, practical adjustments to daily living could lead to an overall lifestyle improvement.

A clear starting point in lifestyle modification is changing dietary patterns. Recent changes to Canada’s Food Guide and other similar guides in other jurisdictions recognize that dietary patterns that are high in added sugar increase the risk for chronic disease (14). Currently, children and adolescents report the highest intake of free sugars of any age group in Canada (15). Strikingly, while the WHO and Canada Dietary Guidelines both recommend that consumption of free sugar does not exceed 10% total caloric intake (16,17), over 80% of Canadian youth 9–18 years of age have reported diets that exceed this sugar intake guideline (15). Sugar-sweetened beverages (SSBs) are any beverage that contains added sugars or have sugar-containing sweeteners added during production, typically including soft drinks, some juices, and energy drinks. Some definitions also included beverages like 100% fruit juices and sweetened milk, like chocolate milk, while other definitions exclude these beverage types. For the purposes of this review, we are using the Health Canada definition of “sugary drinks” as our definition of SSBs (15). These include the following beverage types: “soft drinks, fruit-flavoured drinks, 100% fruit juice, flavoured waters with added sugars, sport and energy drinks, and other sweetened hot or cold beverages, such as iced tea, cold coffee beverages, sweetened milk, and sweetened plant-based beverages” (15).With this definition, 40% of free sugar intake by Canadians was accounted for by the consumption of SSBs (15). Therefore, it is important to investigate any links between the consumption of SSB and children and adolescents who develop obesity, as this could serve as a precise and simple target for weight management initiatives in youth.

Technological advances over the past several decades have made screened products (e.g., smartphones, tablets, computers, gaming consoles, and TV) much more accessible. As a result, total leisure screen use is increasing and contributing to sedentary behaviour and worse sleep patterns (18,19). The WHO has recommended that preschool-age children’s screen time does not exceed one hour per day, as they stress the importance of establishing good patterns of physical activity from a young age (20). As noted by Mark et al. (21), in the early 2000s, the American Academy of Pediatrics recommended limiting children’s screen time to 2 h per day or less (22) and the Canadian Paediatric Society released similar guidelines in May 2003, recommending television time does not exceed 2 h per day (23). The Canadian Paediatric Society updated screen time recommendations for pre-school children (ages <5 years) in November 2022 (24) and published updated advice for healthcare professionals and parents for school-aged children and adolescents in September 2019 (25). Unfortunately, Canadian youth reportedly spending approximately 150% of these recommended times on electronic screens (26). In addition, a 2009 study reported approximately 30% of a sample of American youth spending more than 2 h a day watching television, with the majority of children aged 6–11 years spending 2.5–4 h on digital devices or television (27). Finally, a recent American survey of over 11,000 children and adolescents who are overweight reported that the number of youths meeting the US guideline of <2 h per day of recreational screen time decreased from 44% to 30% from 2018 to 2022 (29)

## AIM AND OBJECTIVES

Firstly, this review will summarize previous research findings regarding individual lifestyle factors (sleep adequacy, SSB consumption, and screen time) and obesity in children and adolescents. Methodological considerations that should be considered by future studies will be highlighted. We will also discuss direct interactions between sleep adequacy, SSB consumption, and screen time, and how they may influence obesity. We aim to delineate how these three modifiable lifestyle factors could be used to target both prevention and treatment of excess weight gain in children and adolescents.

### Sleep adequacy

A simple and common way to operationally define an individual’s sleep adequacy is by its quantity, and the evidence for the link between decreased sleep duration and increased risk of obesity in youth is growing (30,31). As seen in Table 1, previous meta-analyses have reported an association between short sleep duration and obesity in youth and adolescents (32–35). However, though sleep duration is a straightforward way to investigate sleep adequacy in research, the quantity of sleep required changes by age, introducing some complexity particularly in longitudinal studies (35). Furthermore, sleep adequacy encompasses many other components of sleep: time spent in particular sleep stages, such as slow-wave sleep (the deepest sleep phase), sleep fragmentation (number of awakenings or disturbances), sleep efficiency (proportion of time in bed spent asleep), and sleep latency (ability to initiate sleep/the amount of time it takes to fall asleep) (36). A literature review and meta-analysis by Fatima et al. reported a weak association between a wider encompassment of sleep problems (e.g., low duration and poor quality) and obesity in children, adolescents, and young adults (36). Many of the studies included in this analysis were cross-sectional; therefore, the authors suggest that more longitudinal studies be conducted to investigate the strength of sleep-obesity associations (36).

**Table 1.** Summary systematic reviews and meta-analysis of sleep duration and obesity studies in children and adolescents

Study	Number of included studies	Total participants	Age range (years)	Conclusion	Pooled data (95% CI)
Zhang et al. (2017)	12 PCS	44,200	0–19	Shorter sleep duration more likely to be obese	RR = 1.45 (1.14, 1.85)
Ruan et al. (2015)	25 PCS	56,584	0–19	Shorter sleep duration more likely to be obese	OR = 1.76 (1.39, 2.23)
Cappuccio et al. (2008)	13 (Individual designs not reported)	30,002	2–20	Shorter sleep duration more likely to be obese	OR = 1.89 (1.46 to 2.43)
Chen et al. (2008)	17 (3 cohort, 12 CS, 2 CC)	Not reported	0–18	Shorter sleep duration more likely to be obese	OR = 1.58 (1.26, 1.98)

Abbreviations: *CC case-control; CI confidence interval; CS cross-sectional; OR odds ratio; PCS prospective cohort studies; RR relative risk*.

Notes: Cappuccio et al. (2008) collected data on children and adults; however, we report only on their analysis on children. For full definitions of short sleep, please refer to the primary references in this table.

Although previous meta-analyses and reviews have been reporting an association between sleep adequacy and obesity, individual studies within these reports did have some varying findings (32–35). For this reason, it is important to delineate the most appropriate methodology for future studies. There is indeed evidence that sleep quality may influence weight independently of sleep duration, indicating that measuring only one aspect of sleep may not be sufficient when investigating its association with obesity (30,36). The Pittsburgh Sleep Quality Index, a self-report questionnaire of sleep habits that includes measures of both sleep duration and quality, could serve as a useful tool (36,37). Nonetheless, it has been noted that individuals typically overestimate sleep (30), and furthermore, children may not be capable of accurately self-reporting their sleep on such a measure (38). For this reason, incorporating an objective measure of sleep, such as actigraphy, maybe a good practice. Actigraphy involves the use of a wrist-worn device that tracks movement to estimate sleep patterns, offering researchers a non-invasive and cost-effective way to gather sleep data over extended periods in natural environments. This approach provides valuable insights into sleep-wake cycles and is particularly useful for detecting variability in habitual sleep behaviours. However, actigraphy also presents some challenges, such as potential inaccuracies in distinguishing between wakefulness and quiet rest, as well as the need for participant compliance in wearing the device consistently. Despite these limitations, actigraphy remains a widely used tool that can complement subjective measures like sleep diaries, enhancing the reliability and depth of sleep research.

### Obesity in childhood and adolescence

The way in which obesity is defined and measured is another important consideration for all studies investigating weight. Body mass index (BMI) is often used as an objective quantification for obesity, with the condition defined as a measurement of 30 kg/m^2^ or greater on this scale (39). The WHO recommends defining obesity in children aged 5–19 using deviation from standard curves due to growth and development by sex and age (1). Drawbacks to BMI include that it may overestimate body fat for those with high amounts of lean muscle mass (40) and it may have a low sensitivity rate (41). Furthermore, the health risks that arise from obesity are dependent on characteristics of the adipose tissue, such as where it is stored (3,42), qualities that are not quantified by BMI (43). Techniques such as sonographic imaging, X-rays, computed tomography, etc., may be powerful in differentiating between fat types (44) but, unfortunately, this equipment may not always be accessible to researchers or community healthcare providers. Waist circumference is a more accessible measure that correlates with negative health outcomes (44–46), which may therefore complement BMI in future studies to obtain accurate findings applicable to the improvement of health in children and adolescents. The gold standard approach for measuring waist circumference is used across many jurisdictions, including Canada, and the guidelines for use, how to measure, and assess the measurements for adults and children through to the elderly have been reported in the Canadian context (47,48).

### Sleep and energy balance

There are several theories for the potential association between sleep and obesity. As noted by Ogilvie and Patel (30), short sleep duration could lead to increased food intake (49). This may simply be due to the increased time spent awake and opportunity for eating or by individuals increasing food intake in an attempt to counteract symptoms of fatigue (30,31,50,51). It has also been suggested that shorter sleep duration leads to increased energy intake due to changes in hormones that regulate hunger and metabolism, such as leptin, ghrelin, cortisol as well as changes in insulin sensitivity (31,52–56). Another study found that sleep restriction resulted in consumption of higher glycemic index foods, higher glycemic load, and an overall trend towards increased consumption of total calories by children and adolescents (57). Moreover, regardless of the quantity of food items consumed, there is also evidence that inadequate sleep could lead to less nutritious food choices (28,33,57). The increased caloric intake resulting from any of these scenarios would contribute to a positive energy balance, creating opportunities for weight gain.

Subsequent modification of activity levels due to inadequate sleep may also be a mechanism by which sleep patterns manifest in the obesity epidemic. Specifically, getting less sleep or poorer quality sleep leads to fatigue, which may increase sedentary activity (28,33,34,51,58,59). Interestingly, St. Onge and co-authors reported that energy expenditure may increase under conditions of restricted sleep due to increased movement time and greater expenditure by thermoregulation (55). However, calculations indicate that the increase in energy intake following restricted sleep is larger than the increased expenditure, which would indicate a positive energy balance and potential weight gain (55). Further research to confirm this, including long-term studies to determine the stability of this imbalance, is needed (55). It is also important to consider that participation in structured physical activity, with the intention to improve health and fitness, could be hindered by the feelings of fatigue that accompany sleep struggles (34,51).

## SSB CONSUMPTION

### Previous research: SSB consumption and obesity

A considerable amount of research has investigated if there is an association between SSB consumption and obesity, with recent research reviewing, analyzing, and summarizing previous systematic reviews and meta-analyses. A synopsis of these, as well a recent review, is found in Table 2. In general, authors reported an apparent positive association between the consumption of SSB and obesity in children and adolescents (60–62). On the other hand, a review of meta-analyses encompassing solely randomized controlled trials by Nissensohn et al. reported that evidence was not sufficient to draw a conclusion. This shines light on the possibility that confounding variables, such as a general poor-quality diet, may be affecting results in observational studies (60,63,64). Accordingly, Luger et al. (62), acknowledged that their findings of a potential positive association are unable to be extended to causal inferences due to a lack of included interventions. However, it is also important to note that the review by Nissensohn et al. included meta-analyses that had adult participants; this inclusion of an older population introduces another potential reason for the contrasting conclusion (63).

**Table 2.** Previous research related to SSB consumption and obesity in children and adolescents

Study	Design	Number of included studies	Age range (years)	Conclusion
Luger et al. (2017)	Review	20 (17 cohort, 3 RCT)	Children	Evidence suggests positive association with obesity
Nissensohn et al. (2017)	Review of meta-analyses	6 meta-analyses (Including RCTs)	Children, adolescents, and adults	Evidence not sufficient to reach conclusion
Bucher Della Torre et al. (2016)	Analysis of methodological quality	32 (29 cohort, 3 RCT)	<18 years	Majority of studies with strong methodology reported positive association
Keller & Bucher Della Torre (2015)	Review of reviews	13 reviews and meta-analyses (Including longitudinal, intervention, and CS studies)	0.5–19.0 years	Majority reported positive association but discrepant results present

Abbreviations: *CS cross-sectional; RCT randomized control trials; SSB, sugar-sweetened beverages*.

Note: For the actual definition of SSB in each study please refer to the primary reference in this table.

It is important to note that many of the authors of studies that reported a likely association between SSB consumption and obesity drew attention to discrepancies between, and limitations within, individual studies. The lack of a consensus on the definition of SSB has been acknowledged as one major methodological drawback in these studies (60,61). While it is generally accepted that sports drinks, energy drinks, sodas/soft drinks, coffee/tea with added sugar, drink crystal-type drinks, juices with added sugar, and alcohol should be included in the category due to the addition of free sugars during manufacturing or preparation for consumption (60). Controversy arises, however, when considering if 100% fruit juices, without added sugar, should be included in the category of SSB. 100% fruit juices contain natural free sugars, as described in the WHO’s definition of free sugars: “Monosaccharides and Disaccharides added to foods by the manufacturer, cook, or consumer, plus sugars naturally present in honey, syrups, fruit juices and fruit juice concentrate” (17). In fact, simple sugars are the main calorie source in 100% juices and these beverages were found to be the fourth top source of free sugar in Canadian and North American diet’s (15,16,65,66). Additionally, sweetened dairy products, like chocolate milk, are also often not classified as SSB. The argument that is made for not classifying sweetened milk as SSB centres on the fact that dairy products are a source of protein, calcium, and other nutrients and therefore different from other sweetened beverages. In the Canadian context, this was evident when the Newfoundland and Labrador government excluded “chocolate milk” from the 2022 SSB tax implementation (67). For these reasons, it is important to consider that consumption, especially in large amounts, of these 100% juices and sweetened milk may have negative health outcomes.

### The role of SSB consumption in energy balance and sleep

Indeed, the concern for obesity from SSB consumption arises due to the high caloric content of the drinks compared to other liquid alternatives. According to the energy balance theory discussed previously, drinking such beverages without a decrease in other caloric intake or an increase in energy expenditure would result in weight gain (65). Give that SSBs are calorically dense and often lack the satiating effects of solid foods, they can lead to passive overconsumption of calories. Unlike solid foods, SSBs do not seem to trigger the same satiety signals, leading to reduced compensation in total energy intake and thus, greater risk for positive energy balance and weight gain (66). Therefore, limiting consumption or substituting for a lower calorie alternative would decrease energy input, making individuals less likely to be in the positive energy balance state that would lead to weight gain.

Consumption of SSBs has also been associated with poor sleep quality (69), which is concerning due to the detrimental effects inadequate sleep on energy balance as discussed previously (68). Adding to the complexity of the relationship between SSBs and sleep quality are varying effects based on beverage type. For example, Chaput et al. reported that children consuming cola or other sugar-sweetened soft drinks were less likely to meet sleep recommendations (9–11 h per night for 9–11 year olds) (70). One potential explanation for reduced or inefficient sleep is consumption of SSBs caffeine content in energy and sports drinks, as well as sweetened teas and coffees (60). However, Chaput and colleagues reported a higher likelihood of achieving sleep recommendations for children consuming sports drinks of fruit juices with no impact of energy drinks (known to contain high levels of caffeine) (70). It is commonly known that caffeine is a stimulant, which increases alertness, therefore more research is needed in this area to determine the impact of SSB beverage type on sleep duration and quality.

Another contributing factor to poorer sleep quality with increased consumption of SSBs may be a displacement of food and beverages beneficial to sleep. For example, St. Onge et al. suggested that milk products may have sleep-promoting effects (55), while consumption of SSBs has been reported to be associated with a subsequent decreased consumption of milk (65,71,72). The tendency of SSBs to replace nutrient-dense beverages like milk and other dairy products could be explored in more detail. Displacing milk with SSBs may not only reduce sleep duration and quality but also lower the intake of essential nutrients such as amino acids, calcium, magnesium, and vitamin D, which are linked to sleep health as well. Adding further complexity to this dairy-related discussion is the rise in consumption of non-dairy alternatives. Given how new this beverage category is and how fast it is growing in both consumption and products available to consumers, it will surely begin to add noise to the data related to SSBs and sleep health.

There is considerable discrepancy in the scientific literature regarding the effects of SSBs on sleep health in children. Research findings vary widely, with some studies highlighting negative associations, particularly with caffeinated beverages, while others find no clear impact or even potential neutral effects based on beverage type. These variations suggest that the type of SSB consumed may play a critical role in determining its impact on sleep, emphasizing the need for more nuanced research that considers both the beverage category and individual differences in consumption patterns.

## ELECTRONIC SCREEN TIME

### Previous research: electronic screen time and obesity

As the accessibility of electronic screen time is increasing, it is important to consider the implications this may have on children and adolescents’ lifestyle and overall health. Table 3 summarizes previous research regarding screen time and obesity in this age group. Findings from one review and one meta-analysis provide evidence that increased electronic screen time may be associated with or lead to obesity (73–75). However, one review that specifically analyzed video game use and obesity reported inconsistent findings (74). This suggests that other forms of screen time, such as watching television, may play a larger role in the obesity epidemic. Following this idea, other studies have also reported poorer diet during television watching (76). These studies reinforce the concept that eating while engaged with electronic screen time may reduce mindfulness of satiety cues while eating. The specific forms of screen time exposure most greatly associated with obesity require further investigation, Kracht et al. called for more prospective cohort studies (74).

**Table 3.** Previous research summarizing findings regarding screen time usage and obesity in children and adolescents

Author and Year	Design	Included studies	Age range (years)	Conclusion	Pooled data (95%)
Fang et al. (2019)	Meta-analysis	16 (13 CS, 1 cohort, 1 case-control, 1 longitudinal)	<18	Spending > 2 HPD on screen associated with greater obesity risk	OR = 1.67 (1.48, 1.88)
Kracht et al. (2020)	Review	26 (25 CS, 1 longitudinal)	2.0–18.9	Inconsistent evidence for an association between video gaming and obesity	NA
Robinson et al. (2017)	Review	Discussed longitudinal cohort, observational, and RCTs	Children and Adolescents	Reported screen media exposure leads to obesity	NA

Abbreviations: *CS cross-sectional; HPD, hours per day; NA, not applicable; RCTs, randomized control trials*.

The link between electronic screen time and obesity may be mediated by sleep adequacy and could be explained by increased screen time leading to reduced physical activity and decreased energy expenditure. However, the fact that resting metabolic rate increases as body weight increases (7) highlights an important point for studies that wish to investigate changes in energy expenditure. Specifically, an overall change in resting energy expenditure due to weight gain may confound small decreases in energy expenditure due to less physical activity when measured by some common tools in free-living studies (7). This should be considered by future studies, especially ones with longitudinal designs where weight change is more likely to occur.

### The role of electronic screen time in energy balance and sleep

As screen time usage begins to consume an increased number of hours during the day, particularly during downtime close to bedtime, it is important to consider how screen time can affect children and adolescents’ sleeping patterns. Melatonin is a hormone produced by the pineal gland which helps regulate the sleep-wake cycle by increasing propensity to sleep in low-light conditions (77–79). Evening and night-time exposure to bright light and blue light emitted from devices during screen time may suppress melatonin production, making it more difficult for youth to fall asleep (18). In line with previous discussions, research in this area has highlighted the impact this lack of sleep would have on appetite-regulating hormones and eating schedules (57,75).

**Figure 1.**
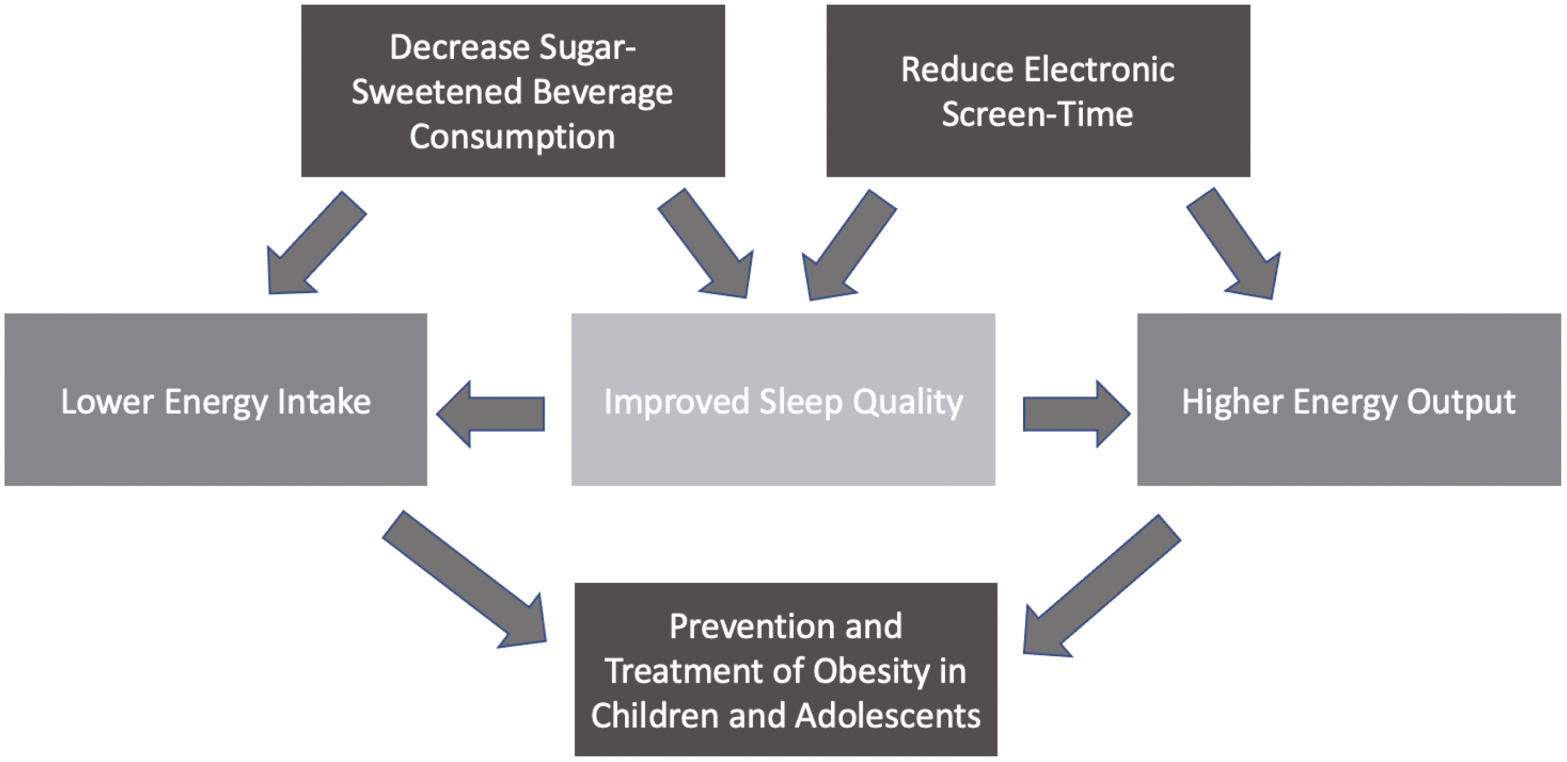
Decreasing SSB consumption may decrease energy intake if the decrease is not offset but an increased caloric intake from another source. It could also improve sleep quality by reducing caffeine intake and increasing consumption of other food and beverages with sleep-promoting effects. Reducing electronic screen time may increase energy expenditure by increasing active time and improve sleep quality by reducing blue light exposure near bedtime. The improved adequacy of sleep will further impact energy balance by promoting healthy hormone levels, decreasing likelihood of eating to counteract fatigue, and increasing intentional exercise. Altogether, SSB consumption, electronic screen time, and sleep quality are modifiable lifestyle factors that could prevent and reduce obesity in children and adolescents.

Furthermore, excessive amounts of screen time may lead to increased sedentary behaviour. Indeed, previous research, outlined by Barnett and colleagues, has reported the highest sedentary time in groups with the most screen time (80). This is important because increased time spent sitting or lying down comes with a decrease in active time and energy expenditure. Therefore, we again see the possibility of positive energy balance and potential for weight gain. The potential effects of modifying this lifestyle factor and others discussed previously, and the suggested interactions between the variables, are presented in Figure 1.

## CONCLUSIONS AND IMPLICATIONS

The obesity epidemic in children and adolescents is of serious concern as it is associated with several negative health outcomes. According to the energy balance theory, to combat obesity, we may decrease energy intake while simultaneously increasing energy expenditure. An important lifestyle target that we have discussed is sleep adequacy: Sleep patterns may indeed mediate the effectiveness of modifying other factors, as well as play an important role in energy balance. To improve the accessibility of and adherence to weight management and obesity interventions, we also aimed to identify simple and practical lifestyle adjustments that will be effective in children and adolescents. Due to the sugar intake levels far above recommended amounts, we first explored the consumption of SSBs as a potential target. In general, past research has reported an association between intake of sugary drinks and obesity. Similarly, with major technological advances over the past several decades and subsequent increases in screen time, we sought to investigate if excessive users are at an increased risk of obesity. Again, past research has indicated that general screen time may be associated with obesity; however, the increased risk could depend on the specific screen time activity. In summary, we suggest that decreasing SSB consumption, reducing electronic screen time, and improving sleep quality would, independently and through interactions with each other, prevent and treat obesity.

While the global reports mostly indicated significant relationships between these modifiable factors and obesity, we acknowledged that many individual studies had inconsistent findings. Following up on this variation, we recommended methodological considerations for future research. Specifically, obesity definitions should attempt to differentiate between forms of mass, measures of sleep adequacy should include other aspects of sleep quality besides solely focussing on sleep duration, and an objective measure of sleep should be included if possible. Any self-report measures should be mindful of young age groups and how their communication efficiency could limit their reporting. SSB definition should be mindful of the effects of naturally sugary drinks, and energy expenditure measures should consider any weight changes that occur over the course of a longitudinal study design.

There are several ways in which reduction of SSB consumption and screen time may be achieved. Changes to marketing and advertisements show potential for reducing SSB consumption (81). Recommending that parents remove electronic devices from children’s bedrooms could be effective in reducing screen time and simultaneously improving sleep quality (80): found that the number of television’s, computers, gaming consoles, and mobile devices in children’s rooms and the household is positively associated with increased screen time. However, this review has not investigated the most effective ways to intervene in sleep quality, SSB consumption, and screen time, and those seeking to implement our recommendations should seek this information in other research.
